# NFSA-DTI: A Novel Drug–Target Interaction Prediction Model Using Neural Fingerprint and Self-Attention Mechanism

**DOI:** 10.3390/ijms252111818

**Published:** 2024-11-03

**Authors:** Feiyang Liu, Huang Xu, Peng Cui, Shuo Li, Hongbo Wang, Ziye Wu

**Affiliations:** School of Information, Guizhou University of Finance and Economics, Guiyang 550025, China; feiyang@mail.gufe.edu.cn (F.L.);

**Keywords:** drug–target interaction prediction, drug discovery, graph neural network, neural fingerprint, self-attention, attention pooling, interpretability

## Abstract

Existing deep learning methods have shown outstanding performance in predicting drug–target interactions. However, they still have limitations: (1) the over-reliance on locally extracted features by some single encoders, with insufficient consideration of global features, and (2) the inadequate modeling and learning of local crucial interaction sites in drug–target interaction pairs. In this study, we propose a novel drug–target interaction prediction model called the Neural Fingerprint and Self-Attention Mechanism (NFSA-DTI), which effectively integrates the local information of drug molecules and target sequences with their respective global features. The neural fingerprint method is used in this model to extract global features of drug molecules, while the self-attention mechanism is utilized to enhance CNN’s capability in capturing the long-distance dependencies between the subsequences in the target amino acid sequence. In the feature fusion module, we improve the bilinear attention network by incorporating attention pooling, which enhances the model’s ability to learn local crucial interaction sites in the drug–target pair. The experimental results on three benchmark datasets demonstrated that NFSA-DTI outperformed all baseline models in predictive performance. Furthermore, case studies illustrated that our model could provide valuable insights for drug discovery. Moreover, our model offers molecular-level interpretations.

## 1. Introduction

Despite significant advances in basic life sciences and biotechnology, the drug discovery and development process is still limited by slow time and high costs [1]. The average duration for creating a small-molecule medication is approximately 15 years, costing around USD 2 billion [2]. While clinical studies are widely acknowledged as a crucial component of drug development, the greatest potential for time and cost savings lies in the earlier discovery stage [3,4]. The prediction of drug–target interaction (DTI) plays a pivotal role in guiding research and development efforts, making it an essential part of the drug discovery process [5,6].

Recently, significant advancements in drug–target interaction prediction have been achieved due to rapid progress in deep learning (DL)-based approaches [7,8,9,10]. Many of these approaches seamlessly integrate the drug chemical space, protein sequence, and interaction information into a comprehensive end-to-end framework [11,12]. They approach DTI prediction as a binary classification task and utilize various deep encoding and decoding modules, such as graph neural networks [13,14,15], deep neural networks [16,17], or transformer architectures [18,19], to make predictions. With the advancements in deep learning techniques, these models can automatically learn data-driven representations of drugs and proteins from extensive DTI data instead of solely relying on predefined descriptors.

However, the current DL-based models, despite exhibiting decent performance, are subject to two limitations. One limitation arises from the predominant focus on local feature extraction, neglecting the acquisition of global representation [13,14,16]. The prediction of drug–target interaction necessitates comprehensive consideration of various aspects pertaining to receptor and ligand information [20]. Therefore, focusing only on local features tends to limit the modeling power and forecasting performance of the model. The second limitation arises from the inability to explicitly model and learn the local key interaction sites between the drug molecule and target protein. This limitation impedes the ability to provide a more intuitive and molecular-level interpretability of the mechanisms underlying drug–target interaction.

In recent years, the combination of molecular fingerprinting and deep learning models has further improved the performance of models in dealing with complex molecular structures [21]. For example, Zhu et al. [17] proposed a fingerprint-embedding framework for drug–target binding affinity prediction (FingerDTA), which uses convolutional neural networks (CNNs) to extract local patterns and utilizes fixed fingerprints to characterize global information. Lee et al. [16] proposed a novel model called DeepConv-DTI, which captures the local residue patterns by convolving amino acid subsequences of various lengths, and it uses a fully connected neural network to encode the fixed ECFP4 [22] drug fingerprint. On the one hand, these traditional fingerprints are usually generated based on the local structure of the molecule (such as specific atoms or chemical substructures) and rely on predefined rules, so they cannot effectively capture the overall topology of the molecule [23]. In contrast, the neural fingerprint [24] based on a graph neural network can gradually integrate local information into global information by recursively aggregating the drug features of all nodes. On the other hand, the limitation of CNN as the protein encoder is that the dependencies between distant amino acids in the protein sequence cannot be captured well [25]. Fortunately, the self-attention mechanism can assign different weights to each position in the sequence, allowing it to capture dependencies between positions more effectively [26]. This offers the potential to enhance CNN’s capability in capturing the global information within the sequence.

Building on the advantages of neural fingerprints, Duvenaud et al. [24] integrated them into graph neural networks (GNNs) to propose the neural fingerprint graph neural network (NFGNN). Originally designed for predicting molecular properties, NFGNN has been shown through comparative experiments by Feldmann et al. [27] to enhance the generalization ability of molecular property predictions, demonstrating robust performance even for molecules that differ from the training set. Recently, the application of NFGNN in DTI prediction has begun to make progress. For instance, Joshy et al. [28] employed NFGNN in the search for drugs to treat orphan diseases, validating its effectiveness. However, the application of NFGNN in DTI prediction is quite scarce. The full potential of NFGNN in this area remains to be further explored.

In recent years, the self-attention mechanism has increasingly been incorporated into prediction models in the DTI field due to its advantages [29]. For instance, compared to DrugBAN developed by Bai et al. [14], CAT-DTI proposed by Zeng et al. [15] has introduced further innovations in the protein encoder module. They integrate the transformer architecture with CNN, enhancing the feature extraction capabilities of CNNs through the self-attention mechanism. Although CAT-DTI demonstrates superior performance compared to existing baseline models, the combination of these two architectures can complicate the training process and hyperparameter tuning. Furthermore, CAT-DTI does not adequately capture local site-specific interactions in drug–target pairs, which limits its interpretability. Therefore, further research is needed to explore the scientific integration of self-attention mechanisms into prediction models and to improve model interpretability.

Therefore, in order to address the aforementioned limitations and explore potential areas for improvement, we propose a drug–target interaction prediction model (NFSA-DTI) based on neural fingerprint and a self-attention mechanism. In conclusion, the key contributions of our work can be summarized as follows:

• We propose an enhancing self-attention convolutional module (ESACM) that utilizes the self-attention mechanism to enhance CNN’s ability in capturing the long-distance dependencies between the subsequences in the target amino acid sequence. ESACM enables the protein encoder to comprehensively calculate the impact of subsequences at different positions on the target sequence.
• We have successfully applied the neural fingerprint graph neural network (NFGNN) to the drug–target interaction prediction task and verified its effectiveness. In contrast to fixed fingerprints, which require extensive lookup tables to uniquely encode all possible molecular structures, neural fingerprints can encode all molecular structures using trainable parameters.
• We propose the integration of an attention pooling method into the bilinear attention network and demonstrate its effectiveness. This pooling method can assign weights to each element in the input matrix based on its importance, thereby augmenting the model’s learning ability of the local pivotal binding sites within the drug–target pair.

## 2. Results and Discussion

### 2.1. Model Performance Comparison

We compared NFSA-DTI with seven baseline models in three benchmark datasets. The results of the comparative experiments are presented in Table 1.

In terms of AUROC, AUPRC, and accuracy, NFSA-DTI was better than the baseline models according to the results in Table 1. In the BindingDB dataset, compared with the currently best-performing CAT-DTI, AUROC, AUPRC, and accuracy of NFSA-DTI were improved by 0.5, 1.0, and 1.1 percentage points, respectively. We believe that the stronger global feature extraction capability, as well as the introduction of multiple attention mechanisms, motivated NFSA-DTI to perform better than CAT-DTI. However, the performance gap between NFSA-DTI and CAT-DTI in the BioSNAP dataset was not particularly obvious. This phenomenon may be caused by the fact that on a balanced and data-heavy BioSNAP dataset the model may be more prone to overfitting during training because it must simultaneously process a large number of samples from various classes, and the features between different classes may interfere with each other, thus affecting the overall performance. To address this issue, we modified the number of neurons per layer in both the NFGNN and CNN models, as well as adjusted their dropout rates, to reduce the risk of potential overfitting. These modifications are intended to further narrow the performance gap with the CAT-DTI model in terms of AUROC and AUPRC.

In terms of sensitivity and specificity, NFSA-DTI did not show complete advantages over the baseline models. This may be because the model tended to predict a certain class of samples, thus inevitably sacrificing a part of the sensitivity to the minority class or the specificity to the majority class. However, the model performed well on AUROC and AUPRC, which indicated that the model could still effectively distinguish between positive and negative samples in general. Overall, NFSA-DTI outperformed all baseline models under most evaluation metrics, thus demonstrating the effectiveness of our proposed model.

### 2.2. Ablation Study

**Figure 1:**
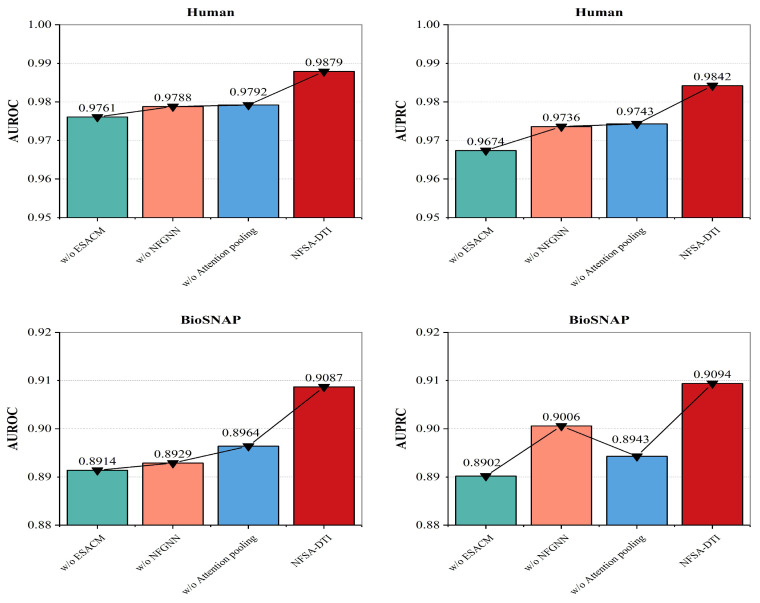
Ablation study on the Human and BioSNAP datasets.

In order to verify the influence of each innovation module in NFSA-DTI on the model’s performance, we designed several model variants to conduct ablation experiments. The results are shown in Figure 1.

The three ablation models are explained as follows:

(1) w/o ESACM: Protein encoder exclusively employs a three-layer CNN without the addition of the self-attention enhancing unit (others same as NFSA-DTI).

(2) w/o NFGNN: Drug encoder employs a three-layer GCN instead of a three-layer NFGNN (others same as NFSA-DTI).

(3) w/o Attention pooling: Sum pooling is used for the final pooling of the bilinear attention network instead of attention pooling (others same as NFSA-DTI).

According to the results in Figure 1, compared with w/o ESACM, NFSA-DTI achieved a 1.18 percentage point increase in AUROC and a 1.68 percentage point increase in AUPRC on the Human dataset, and a 1.73 percentage point increase in AUROC and a 1.92 percentage point increase in AUPRC on the BioSNAP dataset. This is because, compared with the pure use of CNNs as the protein encoder, ESACM could use the self-attention mechanism to effectively enhance the ability of CNNs to capture the long-distance dependencies between the subsequences in the target amino acid sequence, thus further improving the performance. Compared with w/o NFGNN and w/o Attention pooling, NFSA-DTI also had relatively obvious advantages, which also indicated that compared with using GNN alone as the drug encoder, NFGNN used the neural fingerprint encoding the entire molecular structure as an additional global information supplement. In addition, compared with the fixed fingerprint, the neural fingerprint could be obtained by parameter learning and had more efficient retrieval advantages. Compared with sum pooling as the final pooling operation of the bilinear attention network, attention pooling could enhance the model’s ability to learn the local important binding sites in a drug–target pair.

### 2.3. Case Study

In order to verify our model’s ability to discover new drugs and new target proteins, we constructed a new test dataset from the DrugBank online database, consisting of drug–target pairs absent in the BindingDB dataset, and we used the model trained on the BindingDB dataset to conduct the test. Taking Vascular Endothelial Growth Factor Receptor 2 and Nortriptyline as examples, according to the interaction probability score *p*, the predicted top 10 drug and target protein candidates are shown in Table 2 and Table 3, respectively.

As can be seen in Table 2 and Table 3, our model successfully predicted three drugs and two proteins (marked in bold) that could be validated in the DrugBank online database and two drugs and four proteins (marked with underline) that could be validated in the TTD [36] online database. The pharmacological effects of other drug–target pairs in the above two tables were currently unknown in the DrugBank and TTD databases and need to be further verified. It is worth mentioning that the drug candidate or target protein candidate with the highest prediction score in the table was consistent with the ranking recorded in the DrugBank database.

Based on the unknown drug–target interaction pairs mentioned above, researchers can discover new drug candidates that may have therapeutic potential, especially for those diseases that have not been fully studied [37]. For example, in Table 2, 2-aminobenzimidazole would potentially act on Vascular Endothelial Growth Factor Receptor 2 as a new drug. Moreover, for already approved drugs, this will help to search for their possible new mechanisms of action and new therapeutic applications, thereby helping researchers to explore the potential of these drugs in the treatment of other diseases [38]. Similarly, in Table 3, Nortriptyline may act as a repositioning drug on a new target Alpha-2A adrenergic receptor. Although these unknown predictions require further experimental validation, they provide new directions and possibilities for drug research.

### 2.4. Interpretability Analysis

**Figure 2:**
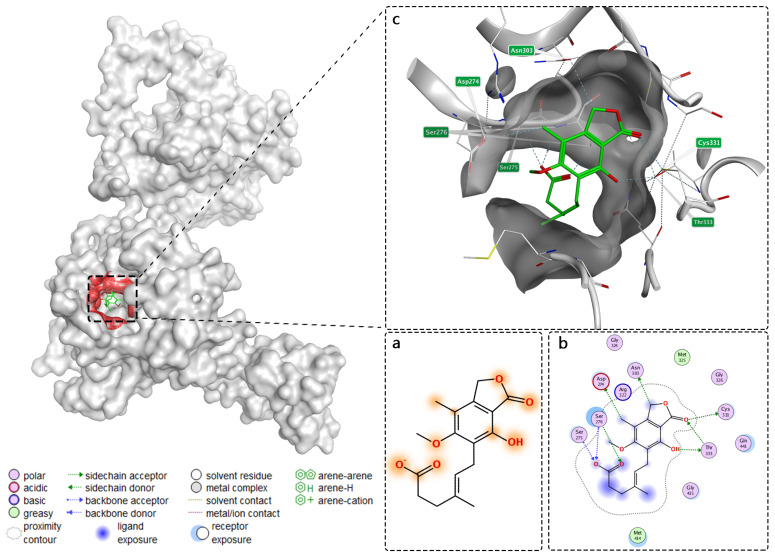
(**a**) The 2D visualization result of mycophenolic acids obtained from NFSA-DTI. The orange highlights indicate possible local binding sites, with darker color and larger area indicating greater likelihood. (**b**,**c**) The 2D and 3D diagrams of the interaction between mycophenolic acid and inosine monophosphate dehydrogenase from the PDB online database, drawn by the software Molecular Operating Environment (MOE 2019.0102) [40].

Another advantage of NFSA-DTI is that it provides molecular-level interpretations essential for drug design efforts. For example, in the Human test dataset, we randomly selected a drug–target interaction pair and plotted the 2D visualization result of its drug molecule as shown in Figure 2a, and we further verified our result by using the Protein Data Bank (PDB) [39] online database, as shown in Figure 2b,c.

According to Figure 2a, our model accurately predicted the involvement of the carboxylate group of mycophenolic acid in receptor binding. This prediction was further validated in Figure 2b, where both oxygen atoms of the carboxylate group were shown to act as hydrogen bond acceptors. One oxygen atom interacts with the back-bone of Ser275 and Ser276, while the other forms a specific interaction with the sidechain of Ser276. Additionally, in Figure 2a, our model predicted that the hydroxyl and methyl groups on the phenyl ring, as well as the carbonyl oxygen atom, are involved in receptor binding. This prediction is confirmed in Figure 2b, where the hydroxyl and methyl groups act as hydrogen bond donors, forming interactions with the sidechains of Thr333 and Asp274, respectively. The carbonyl oxygen atom functions as a hydrogen bond acceptor, interacting with the sidechains of Thr333 and Cys331. Unfortunately, the oxygen atom of the epoxy group was mistakenly predicted to be involved in the receptor interaction. Nonetheless, the overall interpretability of our model provides significant reference value.

In Figure 2b, the dark blue ligand exposure area is mostly concentrated near the carboxylate group of mycophenolic acid. In addition, the light blue receptor exposure area is mainly concentrated near Ser276. Combined with the orange area in Figure 2a indicating possible interaction, it can be inferred that the carboxylate group’s oxygen negative ion and the oxygen atom of the carboxylate group are the most important in this interaction. The 3D visualization of Figure 2c, as a supplement to the 2D visualization of Figure 2b, will help researchers analyze the 3D structure of mycophenolic acid and inosine monophosphate dehydrogenase acceptor and identify key pharmacophore and binding site.

## 3. Materials and Methods

### 3.1. Datasets

We evaluated NFSA-DTI using three publicly available DTI datasets: BindingDB, BioSNAP, and Human. Information about them is shown below.

• The BindingDB Dataset [41] is a large drug–target dataset, containing thousands of small molecule drugs and protein targets. The targets also cover different species, but mainly focus on human targets. It is worth mentioning that BindingDB is unbalanced in terms of dataset distribution.
• The BioSNAP dataset, developed by Huang et al. [18] and Zitnik et al. [42] from the DrugBank database [43], is a balanced dataset. It includes verified positive samples as well as an equal number of randomly paired negative samples that have never been encountered before. This dataset considers interactions between small chemical drugs and target proteins, all of which have been experimentally validated via biological experiments or formal pharmacological studies.
• The Human dataset, constructed by Liu et al. [44], is a balanced dataset, incorporating high-confidence negative samples obtained through silicon screening methods.

Table 4 gives the detailed statistics of these three datasets. In the table, Interactions* are defined as the total number of interacting sample pairs and non-interacting sample pairs in the dataset. P2N represents the ratio of the number of interacting sample pairs to non-interacting sample pairs in the dataset.

The positive samples in the three datasets are based on the literature and experimental verification. However, due to the relative scarcity of experimentally verified negative sample data, researchers usually need to adopt different strategies to construct negative sample sets [9,16]. For the Human dataset, based on the existing biological data and chemical characteristics, the researchers used silicon screening computing technology to efficiently screen out the combinations with low correlation as negative samples, so as to improve the confidence of negative samples. Regarding the BioSNAP dataset, using the biological network construction method, the researchers identified pairs of nodes that were not directly connected in the network and randomly selected negative samples from them to ensure the confidence of the negative samples. For the BindingDB dataset, researchers generated negative samples by excluding known drug–target binding pairs and rigorously screened negative samples to ensure that these combinations did not overlap with positive samples.

### 3.2. Baselines

In order to comprehensively evaluate the prediction performance of our model, we chose seven representative baseline models, including two machine learning models (SVM [45] and RF [46]), and five deep learning models (DeepConv-DTI [16], GraphDTA [13], MolTrans [18], DrugBAN [14], and CAT-DTI [15]). Information about them is shown below.

• **SVM**: By learning the optimal hyperplane, the interaction between drugs and targets can be effectively distinguished in the high-dimensional feature space, which has a relatively strong classification ability and good generalization performance.
• **RF**: By integrating multiple decision trees, the interaction between drugs and targets is predicted in a voting manner, which has strong anti-noise and robustness. It performs well when dealing with high-dimensional data, but may be vulnerable to uneven feature importance.
• **DeepConv-DTI**: By using a convolutional neural network, amino acid subsequences of various lengths are convolved to capture local residue patterns, and a fully connected neural network is used to encode the fixed ECFP4 drug fingerprint. It outperforms previous models based on protein descriptors.
• **GraphDTA**: By representing drugs as graphs and using the graph neural network to predict the affinity of the drug to the target, it can effectively process the topological structure data of drug molecules and improve the prediction accuracy.
• **MolTrans**: By introducing the self-attention mechanism of the transformer, the drug molecules and protein sequences are embedded into a unified vector space, in order to effectively capture the interaction characteristics between them. It has high flexibility and performance in dealing with complex molecular relationships.
• **DrugBAN**: By introducing a bilinear attention network, the interaction strength between the drug and the substructure of the target will be embedded into the bilinear attention matrix for downstream prediction tasks. It can better capture the local feature correlation and improve the performance of the model.
• **CAT-DTI**: By combining the graph convolutional neural network, a transformer architecture, and the cross-attention mechanism, it can effectively capture the information of drug and target sequences and improve the prediction performance. It has an advantage when dealing with long target sequences and is better able to model complex interactions.

### 3.3. Metrics

In our experiment, the AUROC (area under the receiver operating characteristic curve) and AUPRC (area under the precision-recall curve) serve as primary metrics for assessing model classification performance. AUROC measures the model’s ability to discriminate between positive and negative samples across different classification thresholds. It considers both the true positive rate (sensitivity) and the false positive rate (1—specificity). A higher AUROC value indicates better discriminative power and overall performance. AUPRC, on the other hand, focuses on the trade-off between precision and recall. It measures the model’s ability to correctly identify positive samples while minimizing false positives. In addition, we adopted accuracy, sensitivity, and specificity under the optimal F1 score threshold as additional evaluation metrics.

### 3.4. Implement Details

**Figure 3:**
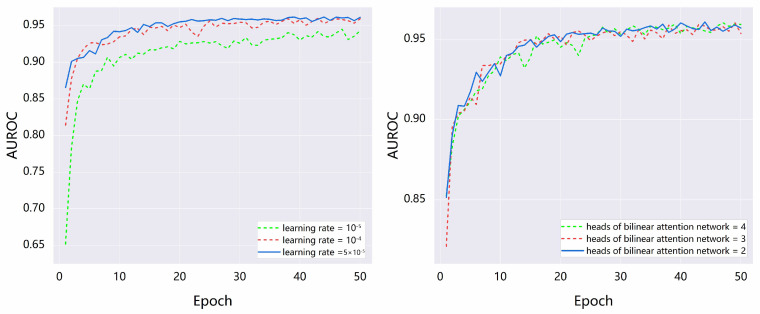
Learning curves of NFSA-DTI when changing some hyperparameters on the validation set of the BindingDB dataset.

To optimize the implementation of NFSA-DTI, we randomly split each dataset into train/validate/test sets at a ratio of 0.7/0.1/0.2. The test set is employed to evaluate the model’s final performance and is entirely independent of the training and validation processes, thereby ensuring the objectivity and accuracy of the evaluation results. Compared to the 7:2:1 division, the 7:1:2 division enhances the reliability of the model’s final evaluation. We used the Human dataset to train the NFSA-DTI model and determined the initial combination of hyperparameters. Subsequently, we fine-tuned the parameters and trained NFSA-DTI. When NFSA-DTI exhibited superior performance on the validation set of each dataset, we determined the optimal values of the hyperparameters. The partial learning curves for the BindingDB validation set are depicted in Figure 3, illustrating the influence of varying certain hyperparameter values. The figure demonstrates that the optimal performance of the model is achieved when the learning rate of the optimizer reaches 5 × 10^−5^ and the number of attention heads in the bilinear attention network is set to 2. The main hyperparameters configuration is shown in Table 5. In this study, we conducted our experiments using Python 3.8 as the programming language. The deep learning framework used is PyTorch version 1.12, and we trained the model using an RTX 4090 24 G, accelerating the computation using NVIDIA CUDA version 11.6.

### 3.5. Methods

**Figure 4:**
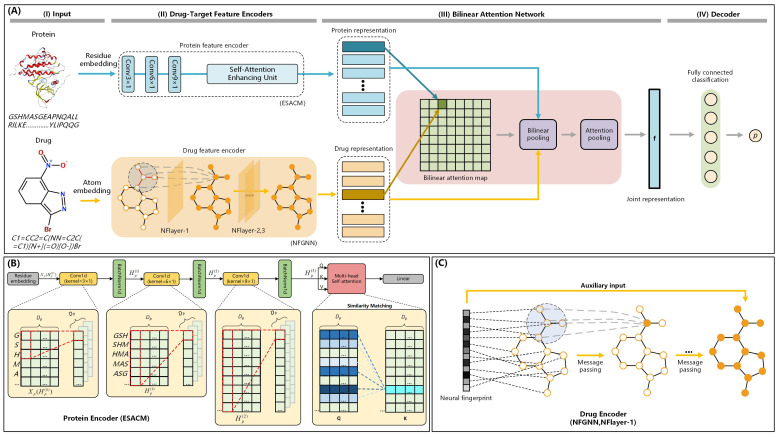
(**A**) The framework of NFSA-DTI. It includes four stages. (I) The target protein’s amino acid sequence is transformed into a feature matrix, while the drug molecule’s SMILES is processed into graph structure data. (II) The 3-layer CNN processes the two-dimensional feature matrix and obtains the protein representation after the self-attention enhancing unit. Correspondingly, the 3-layer NFGNN processes the graph structure data and obtains the drug representation. (III) Interactions between protein and drug representations are computed via a bilinear attention mechanism, thereby producing a bilinear attention map. (IV) The joint representation obtained after pooling will be input to the fully connected layer for computing the prediction score p. (**B**) The framework of ESACM. It comprises three 1D convolutional layers, the corresponding 1D batch normalization layers, a self-attention enhancing unit, and a linear layer. The self-attention enhancing unit consists of two stages. (Stage I) Query, key, and value are computed based on the input matrix, in conjunction with their respective weight matrices. (Stage II) The similarity between query and key is evaluated to derive the attention weights. Subsequently, the output is generated through a weighted summation of the values, utilizing the computed attention weights. (**C**) The flowchart of NFGNN. Firstly, the target node and neighbor nodes surrounding the target node in the molecular graph are integrated and encoded into numerical features. Similar operations are then performed on subsequent nodes to obtain the final neural fingerprint. Subsequently, after the message passing mechanism has completed one iteration of the graph, the neural fingerprint serves as a fixed auxiliary input for updating the graph after each NFlayer.

Figure 4A shows the framework of our proposed NFSA-DTI model.

#### 3.5.1. Problem Formulation and Prerequisites

The objective of DTI prediction is to develop a model *M*, which effectively maps the combined feature representation space P×G of a protein sequence *P* and a drug molecular graph *G*, yielding an interaction probability score p∈[0,1].

The target protein is represented by one amino acid sequence denoted as $P=(a_{1},\ldots ,a_{n})$, where each token corresponds to one of the 23 amino acids. The maximum length of *P* is set to 1200, which is long enough to cover common amino acid sequences while remaining computationally and storage efficient. Amino acid sequences that exceed this threshold are truncated. For amino acid sequences that are not of length 1200, zeros will be padded at the end of the sequence. The amino acid sequences [47] are a standardized representation of proteins that can be expressed and processed in a computable way.

Major current deep learning methods for predicting drug–target interaction typically use the simplified molecular input line entry system (SMILES) [48] as an input representation. The limitation of SMILES lies in its one-dimensional nature, which hinders its ability to capture the intricate spatial arrangement of molecules, potentially resulting in the loss of crucial chemical information. However, in our model, the input SMILES are converted into the corresponding two-dimensional graph structure data *G*. It creates actual nodes and edges based on the drug molecule’s atoms and chemical bonds, with virtual nodes and self-loop edges filling in the rest. Node count is capped at 290.

#### 3.5.2. Protein Feature Encoder

The conventional CNN architecture exhibits certain limitations in handling long-distance dependencies of sequences due to the restricted receptive field of convolution operations [49]. In contrast, the self-attention mechanism enables each position to interact with all other positions within the tensor, granting access to comprehensive information about the entire tensor [50]. Therefore, we propose ESACM to augment model performance. ESACM integrates a self-attention mechanism that enhances the CNNs’ ability to capture the long-distance dependencies between the subsequences in the target amino acid sequence. The framework diagram of ESACM is shown in Figure 4B.

Building upon the concept of word embedding for amino acid sequences by Bai et al. [14], the amino acids are initialized into a learnable embedding matrix $E_{p}\in R^{23\times D_{p}}$, where 23 is the number of amino acid types and $D_{p}$ is the latent space dimension. By looking up $E_{p}$, each protein sequence *P* can be initialized to the corresponding feature matrix $X_{p}\in R^{\Theta_{p}\times D_{p}}$, where the parameter $\Theta_{p}$ represents the maximum permissible length of a protein sequence, which is generally 1200 in our experiment, and each row of the matrix denotes a residue representation in the target amino acid sequence. In the initial convolution layer, the feature matrix $X_{p}$ will be input into the first CNN for one-dimensional convolution to extract amino acid sequence features, and this process utilizes a convolution kernel sized 3 × 1 and a stride of 1, such as “GSHMAS…PQQG” ⟶ “GSH”, “SHM”, “HMA”, “MAS”, …, “PQQ”, and “QQG”. The next two layers keep the same stride as the first, but further enlarge the kernel size to acquire more features of local subsequences. The convolutional operations can be expressed by the following formula:

(1) $$H_{p}^{\left(l+1\right)}=\sigma \left(\mathrm{CNN}\left(W_{c}^{\left(l\right)},b_{c}^{\left(l\right)},H_{p}^{\left(l\right)}\right)\right),$$

where $H_{p}^{\left(l+1\right)}$ is the *l*-th hidden protein representation and $H_{p}^{\left(0\right)}=X_{p}$. σ(·) denotes ReLU(·). $W_{c}^{\left(l\right)}$ and $b_{c}^{\left(l\right)}$ are learnable weight matrices (filters) and bias vector in the *l*-th CNN layer.

The output of the third convolutional layer will subsequently undergo computation in the self-attention enhancing unit. Specifically, the self-attention enhancing unit consists of two stages, corresponding to the following formulas.

Stage I:

(2) $$q_{i,j}^{\left(l\right)}=W_{q}^{\left(l\right)}f_{i,j},k_{i,j}^{\left(l\right)}=W_{k}^{\left(l\right)}f_{i,j},v_{i,j}^{\left(l\right)}=W_{v}^{\left(l\right)}f_{i,j}.$$

Stage II:

(3) $$p_{i,j}=\prod_{l=1}^{N}\left(\sum_{a,b\in N_{m}\left(i\right)}A\left(q_{i,j}^{\left(l\right)},k_{a,b}^{\left(l\right)}\right)v_{a,b}^{\left(l\right)}\right).$$

The specific formula is presented as follows:

(4) $$p_{i,j}=\prod_{l=1}^{N}\left(\sum_{a,b\in N_{m}\left(i\right)}A\left(W_{q}^{\left(l\right)}f_{i,j},W_{k}^{\left(l\right)}f_{a,b}\right)W_{v}^{\left(l\right)}f_{a,b}\right),$$

where $p_{i,j}$ is the value in row *i* and column *j* of the protein representation. ∏ is the concatenation of the outputs of *N* attention heads. $N_{m}\left(i\right)$ represents one column region whose length is the maximum column length *m* of the feature matrix, and its abscissa is *i*. $A(W_{q}^{\left(l\right)}f_{i,j},W_{k}^{\left(l\right)}f_{a,b})$ is the corresponding attention weight with regard to the features within $N_{m}\left(i\right)$. $W_{q}^{\left(l\right)},W_{k}^{\left(l\right)},$ and $W_{v}^{\left(l\right)}$ are the projection matrices for queries, keys, and values, respectively. $f_{i,j}\in H_{p}^{\left(3\right)}$ and $H_{p}^{\left(3\right)}$ denote the output matrix of the third CNN. The attention weight is calculated as follows:

(5) $$A\left(W_{q}^{\left(l\right)}f_{i,j},W_{k}^{\left(l\right)}f_{a,b}\right)=\mathrm{softmax}\left(\frac{{\left(W_{q}^{\left(l\right)}f_{i,j}\right)}^{⊤}\left(W_{k}^{\left(l\right)}f_{a,b}\right)}{\sqrt{d}}\right),$$

where *d* is the feature dimension of $W_{q}^{\left(l\right)}f_{i,j}$.

#### 3.5.3. Drug Feature Encoder

Graph neural network (GCN) [51] primarily updates node representations through local neighborhood information propagation, its ability to model the global structure is relatively limited. NFGNN, a type of neural network proposed by Duvenaud et al. [24], was originally designed for predicting molecular properties and has been proven effective in learning the representation of molecular graphs. Compared with GCN, NFGNN uses the neural fingerprint as an additional global information supplement. Moreover, in contrast to fixed fingerprints that necessitate a substantial number of lookup tables for encoding all possible molecular structures uniquely, neural fingerprints of NFGNN can encode the entirety of molecular structures using trainable parameters. Given the advantages of NFGNN, we therefore introduce NFGNN as the drug encoder of NFSA-DTI.

The flowchart of NFGNN is illustrated in Figure 4C. Specifically, the construction process of the neural fingerprint is represented by the following formulas:

(6) $$r_{a}=g\left(a\right),$$

(7) $$v_{i}=r_{i}+\sum_{j=1}^{N}r_{j},j\in N\left(i\right),$$

(8) $$f=\sum_{i=1}^{M}\left(\mathrm{softmax}\left(\sigma \left(v_{i}H_{h}\right)W_{o}\right)\right),$$

where *a* represents the node. g(·) is the encoding function of node features. $v_{i}$ represents the sum of features about the *i*-th node and its neighbors. N(i) is the set of neighbor nodes of the *i*-th node. $H_{h}$ is the hidden weight, and $W_{o}$ is the output weight. *N* denotes the number of neighbor nodes of the *i*-th node, and *M* denotes the total number of nodes in the graph. σ represents ReLU(·). The subsequent message passing process can be expressed by the following formulas:

(9) $$m_{ij}^{\left(l\right)}=\rho^{\left(l\right)}\left(h_{i}^{\left(l\right)},h_{j}^{\left(l\right)},h_{e_{ij}}^{\left(l\right)}\right),j\in N\left(i\right),$$

(10) $$m_{i}^{\left(l\right)}=\zeta^{\left(l\right)}\left(m_{ij}^{\left(l\right)}|j\in N\left(i\right)\right),$$

(11) $$h_{i}^{\left(l+1\right)}=\phi^{\left(l\right)}\left(h_{i}^{\left(l\right)},m_{i}^{\left(l\right)},f\right),$$

where $\rho^{\left(\cdot \right)}$, $\zeta^{\left(\cdot \right)}$, and $\phi^{\left(\cdot \right)}$ are functions of message construction, message aggregation, and vertex update of the *l*-th layer NFGNN, respectively. $h_{i}^{\left(l\right)}$ is the node feature of the *i*-th node at the *l*-th layer, $h_{j}^{\left(l\right)}$ is its neighbor node feature, $h_{e_{ij}}^{\left(l\right)}$ is the corresponding edge feature, and m is the message.

#### 3.5.4. Bilinear Attention Network

**Figure 5:**
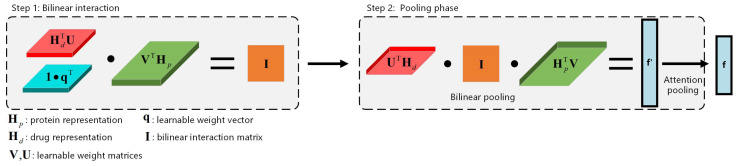
The flowchart of the bilinear attention network. This module consists of two steps. (Step 1) A bilinear interaction matrix is derived through the computation of protein and drug representations. (Step 2) Subsequently, a joint representation is obtained by employing bilinear pooling and attention pooling.

We employ the bilinear attention network proposed by Bai et al. [14] and improve its subsequent pooling operation. Specifically, we replace the summation pooling with an attention pooling. The sum pooling method is relatively straightforward to implement and incurs a low computational cost. However, it lacks the ability to retain the intensity information of features, potentially compromising learning performance. To address this limitation and enhance the model’s capacity in capturing important information between drug substructures and target substructures, we employ an attention pooling approach inspired by Er et al.’s [52] investigation on the importance of sentence components. The attention pooling assigns weights based on the significance of each element in the input matrix, generating an enhanced pooled representation. The enhanced pooled representation promotes the model’s learning of the drug–target interaction modeling process, thereby improving the overall interpretability of the model. The overall flowchart of the improved bilinear attention network is shown in Figure 5.

Given the protein representation and drug representation $H_{p}^{\left(3\right)}=\left\{h_{p}^{1},h_{p}^{2},\ldots ,h_{p}^{M}\right\}$, among them, $h_{p}^{1}=\left\{p_{1,1},p_{1,2},\ldots ,p_{1,e}\right\}$, *…*, $h_{p}^{M}=\left\{p_{M,1},p_{M,2},\ldots ,p_{M,e}\right\}$, and $H_{d}^{\left(3\right)}=\left\{h_{1}^{\left(3\right)},h_{2}^{\left(3\right)},\ldots ,h_{N}^{\left(3\right)}\right\}$ via the protein and drug encoders, where *M* represents the maximum number of target protein substructures encoded by ESACM, *N* represents the maximum number of nodes in the drug molecular graph, and *e* represents the embedding dimension of the CNN. The bilinear interaction matrix $I\in R^{N\times M}$ can be obtained as follows:

(12) $$I=\left(\left(1\cdot q^{⊤}\right)\circ \sigma \left({\left(H_{d}^{\left(3\right)}\right)}^{⊤}U\right)\right)\cdot \sigma \left(V^{⊤}H_{p}^{\left(3\right)}\right),$$

where U and V are learnable weight matrices for drug and protein representations, q is a learnable weight vector, 1 is a fixed all-ones vector, and the symbols ∘ and σ represent the Hadamard (element-wise) product and ReLU(·). The elements in I represent the intensity of interaction between drug–target substructures, indicating their potential binding sites. In order to intuitively understand bilinear interaction, element $I_{i,j}$ can be written as follows:

(13) $$I_{i,j}=q^{⊤}\left(\sigma \left(U^{⊤}h_{d}^{i}\right)\circ \sigma \left(V^{⊤}h_{p}^{j}\right)\right),$$

where $h_{d}^{i}$ is the *i*-th row of $H_{d}^{\left(3\right)}$ and $h_{p}^{j}$ is the *j*-th row of $H_{p}^{\left(3\right)}$, respectively, denoting the *i*-th and *j*-th substructural representations of the drug and protein.

In order to obtain the joint representation $f^{\prime }$, the interaction matrix I is processed by a bilinear pooling layer. Specifically, $f^{\prime }$ is computed as follows:

(14) $$f^{\prime }=\sigma {\left({\left(H_{d}^{\left(3\right)}\right)}^{⊤}U\right)}^{⊤}\cdot I\cdot \sigma \left({\left(H_{p}^{\left(3\right)}\right)}^{⊤}V\right),$$

where the weight matrices U and V are shared with the preceding interaction matrix layer to reduce the number of parameters and mitigate overfitting.

Subsequently, the final joint representation f will be obtained after the attention pooling, which is represented by the following formulas:

(15) $$W_{i,j}=\mathrm{Softmax}\left(b_{a}^{⊤}\tanh\left(U_{a}I_{i,j}\right)\right),$$

(16) $$f=\sum_{i=1}^{N}\sum_{j=1}^{M}\left(W_{i,j}\cdot b_{a}^{\prime }\right)f^{\prime },$$

where $b_{a}$ and $b_{a}^{\prime }$ represent the bias vectors, $U_{a}$ denotes the weight matrix, and $W_{i,j}$ is the attention weight vector.

#### 3.5.5. Fully Connected Classification

The interaction probability is computed by feeding the joint representation f into the decoder, which comprises a single fully connected classification layer followed by a sigmoid function, as follows:

(17) $$p=\mathrm{Sigmoid}(W_{o}f+b_{o}),$$

where $W_{o}$ is the learnable weight matrix and $b_{o}$ is the bias vector.

#### 3.5.6. Backpropagation

The backpropagation process aims to minimize the cross-entropy loss [53] in order to optimize the model’s performance. The formula is presented as follows:

(18) $$L=-\sum_{i}\left(y_{i}\log\left(p_{i}\right)+\left(1-y_{i}\right)\log\left(1-p_{i}\right)\right)+\frac{\lambda }{2}∥\Theta {∥}_{2}^{2},$$

where $y_{i}$ denotes the ground truth label for the *i*-th drug–target pair, $p_{i}$ represents its output probability according to the model, Θ represents all the weight matrices and bias vectors that can be learned, and λ is a hyperparameter used for L2 [54] regularization.

## 4. Conclusions

In this article, we propose a novel DTI prediction model called NFSA-DTI. In the protein encoder, ESACM captures the long-distance dependencies between the subsequences in the target amino acid sequence while extracting the local features of the sequence. In the drug encoder, NFGNN extracts features of the drug molecular graph via a message-passing mechanism, supplemented by the neural fingerprint as global information. In the feature fusion module, the bilinear attention network generates an enhanced pooled representation via attention pooling, improving the model’s ability to learn key local binding sites in the drug–target pair. The experimental results on three benchmark datasets demonstrated that NFSA-DTI outperformed all baseline models in terms of prediction performance. Further case experiments showed that the model has provided valuable insights for drug discovery efforts. Furthermore, the model provides more intuitive interpretability at the molecular level. In the future, we plan to extend our study by using other benchmark datasets.

## Figures and Tables

**Table 1:** Performance comparison with baseline models on the BindingDB, BioSNAP, and Human dataset (**Best**).

Datasets	Model	AUROC	AUPRC	Accuracy	Sensitivity	Specificity
BindingDB	SVM	0.939	0.928	0.825	0.781	0.866
	RF	0.942	0.921	0.880	0.875	0.892
	DeepConv-DTI	0.945	0.925	0.882	0.873	0.894
	GraphDTA	0.951	0.934	0.888	0.882	0.897
	MolTrans	0.952	0.936	0.887	0.877	0.902
	DrugBAN	0.960	0.948	0.904	0.900	0.908
	CAT-DTI	0.960	0.947	0.896	0.884	**0.913**
	NFSA-DTI	**0.965**	**0.957**	**0.907**	**0.908**	0.906
BioSNAP	SVM	0.862	0.864	0.777	0.711	0.841
	RF	0.860	0.886	0.804	0.823	0.786
	DeepConv-DTI	0.886	0.890	0.805	0.760	0.851
	GraphDTA	0.887	0.890	0.800	0.745	0.854
	MolTrans	0.895	0.897	0.825	0.818	0.831
	DrugBAN	0.903	0.902	0.834	0.820	0.847
	CAT-DTI	**0.909**	0.907	0.836	**0.825**	0.847
	NFSA-DTI	**0.909**	**0.909**	**0.839**	0.819	**0.858**
Human	SVM	0.913	0.905	0.838	0.782	0.830
	RF	0.939	0.927	0.866	0.833	0.893
	DeepConv-DTI	0.978	0.982	0.878	0.830	0.938
	GraphDTA	0.965	0.955	0.908	0.912	0.904
	MolTrans	0.981	0.976	0.941	**0.949**	0.939
	DrugBAN	0.981	0.969	0.940	0.938	0.941
	CAT-DTI	0.983	0.976	0.942	0.929	**0.957**
	NFSA-DTI	**0.988**	**0.984**	**0.945**	0.944	0.955

**Table 2:** The predicted top 10 drug candidates for Vascular Endothelial Growth Factor Receptor 2 (P35968) in the newly constructed dataset.

Rank	Drug Name	DrugBank ID	Evidence
1	**Sorafenib**	**DB00398**	Iyer et al. [30]
2	Regorafenib	DB08896	Southan et al. [31]
3	2-Aminobenzimidazole	DB06938	Unknown
4	1-Naphthalenecarboxamide	DB07274	Unknown
5	Ponatinib	DB08901	Unknown
6	**Sunitinib**	**DB01268**	Schoffski et al. [32]
7	Tyrosine Kinase-IN-1	DB05014	Unknown
8	**Lenvatinib**	**DB09078**	Matsui et al. [33]
9	Fostamatinib	DB12010	Unknown
10	RAF265	DB05984	Southan et al. [31]

**Table 3:** The predicted top 10 target protein candidates for Nortriptyline (DB00540) in the newly constructed dataset.

Rank	Protein Name	Uniprot ID	Evidence
1	**Sodium-dependent noradrenaline transporter**	**P23975**	Kim et al. [34]
2	Alpha-2A adrenergic receptor	P08913	Unknown
3	5-hydroxytryptamine receptor 2A	P28223	Southan et al. [31]
4	5-hydroxytryptamine receptor 1A	P08908	Southan et al. [31]
5	5-hydroxytryptamine receptor 1C	P08909	Southan et al. [31]
6	5-hydroxytryptamine receptor 2C	P28335	Southan et al. [31]
7	Alpha-1B adrenergic receptor	P35368	Unknown
8	**Sodium-dependent serotonin transporter**	**P31645**	Vaishnavi et al. [35]
9	Beta-1 adrenergic receptor	P08588	Unknown
10	Muscarinic acetylcholine receptor M1	P11229	Unknown

**Table 4:** Experimental datasets statistics.

Datasets	Drugs	Proteins	Interactions*	P2N
BindingDB	14,643	2623	49,199	0.725
BioSNAP	4510	2181	27,464	1.014
Human	2726	2001	6728	1

**Table 5:** Hyperparameters configuration.

Module	Hyperparameters	Value
ESACM	Initial amino acid embedding dimension	128
	Kernel size	[3, 6, 9]
	Number of filters	[128, 128, 128]
	Heads of self-attention	2
NFGNN	Initial atom embedding dimension	128
	Hidden node dimensions	[128, 128, 128]
Bilinear attention network	Heads of bilinear attention	2
	Bilinear embedding dimension	768
	Attention pooling window size	3
	Attention pooling stride	3
Fully connected decoder	Number of hidden neurons	512
Optimizer	Learning rate	5 × 10^−5^
	Epoch	100
Mini-batch	Batch size	64

## Data Availability

NFSA-DTI and datasets used in this study are available on GitHub at https://github.com/xiaofeinb233/NFSA_DTI (accessed on 20 October 2024).
